# Efficacy of gabapentin for prevention of postherpetic neuralgia: study protocol for a randomized controlled clinical trial

**DOI:** 10.1186/s13063-016-1729-y

**Published:** 2017-01-14

**Authors:** Manuel Rullán, Oana Bulilete, Alfonso Leiva, Aina Soler, Antonia Roca, María José González-Bals, Patricia Lorente, Joan Llobera, PHN group, Martí Cladera, Catalina Comas, María Antonia Mir, Apolonia Cifre, Biel Lliteras, Salvador Gestoso, Antoni Jover, Francisca Bestard, Francisca Comas, Luis López, Rosa Ortuño, Joan Peiro, María Cerdó, Violeta Ramírez, Merce Gutierrez, Rosemary Argüelles, María Dolores Gutierrrez

**Affiliations:** 1 Pollença Health Care Centre, Baleares Health Services (IB-Salut), 07460 Pollença, Spain; 2 Son Espases Hospital, Baleares Health Services (IB-Salut), 07010 Palma, Spain; 3 Primary Care Research Unit of Mallorca, Baleares Health Services (IB-Salut), 07005 Palma, Spain; 4 Instituto de Investigación Sanitaria de Palma (IdISPa), 07010 Palma, Spain; 5 Son Serra-La Vileta Health Care Centre, Baleares Health Services (IB-Salut), 07013 Palma, Spain; 6 Manacor Health Care Centre, Baleares Health Services (IB-Salut), 07500 Manacor, Spain; 7 Calvià Health Care Centre, Baleares Health Services (IB-Salut), 07184 Calvià, Spain

**Keywords:** Postherpetic neuralgia, Gabapentin, Herpes zoster, Prevention, Primary health care

## Abstract

**Background:**

Postherpetic neuralgia (PHN) is a chronic neuropathic pain that results from alterations of the peripheral nervous system in areas affected by the herpes zoster virus. The symptoms include pain, paresthesia, dysesthesia, hyperalgesia, and allodynia. Despite the availability of pharmacological treatments to control these symptoms, no treatments are available to control the underlying pathophysiology responsible for this disabling condition.

**Methods/design:**

Patients with herpes zoster who are at least 50 years old and have a pain score of 4 or higher on a visual analogue scale (VAS) will be recruited. The aim is to recruit 134 patients from the practices of general physicians. Participants will be randomized to receive gabapentin to a maximum of 1800 mg/day for 5 weeks or placebo. Both arms will receive 1000-mg caplets of valacyclovir three times daily for 7 days (initiated within 72 h of the onset of symptoms) and analgesics as needed. The primary outcome measure is the percentage of patients with a VAS pain score of 0 at 12 weeks from rash onset. The secondary outcomes measures are changes in quality of life (measured by the SF-12 questionnaire), sleep disturbance (measured by the Medical Outcomes Study Sleep Scale), and percentage of patients with neuropathic pain (measured by the Douleur Neuropathique in 4 Questions).

**Discussion:**

Gabapentin is an anticonvulsant type of analgesic that could prevent the onset of PHN by its antihypersensitivity action in dorsal horn neurons.

**Trial registration:**

ISRCTN Registry identifier: ISRCTN79871784. Registered on 2 May 2013.

**Electronic supplementary material:**

The online version of this article (doi:10.1186/s13063-016-1729-y) contains supplementary material, which is available to authorized users.

## Background

Herpes zoster (HZ), often called *shingles*, is a common disease characterized by a painful, unilateral vesicular eruption that is caused by reactivation of a dormant varicella zoster virus within the dorsal root or cranial nerve ganglia. The most frequent complication following an acute episode of HZ infection is postherpetic neuralgia (PHN), a chronic and debilitating neuropathic pain syndrome that is refractory to most therapeutic strategies.

PHN is considered a clinically significant problem because it may last for years and because it negatively impacts a patient’s quality of life across all four health domains: physical, psychological, functional, and social. The symptoms associated with PHN are sleep disturbances, mood changes, depression, and anxiety [1].

The authors of a recent systematic review reported that the incidence of HZ infection was between 3 and 12 cases per 1000 patient-years for individuals older than 50 years of age, and that the overall risk of PHN ranges from 5% to more than 30% [2]. Differences in the reported incidence of PHN are due to the lack of consensus on the definition of PHN (i.e., use of different durations of persisting pain from the onset of shingles for a positive diagnosis) and differences in the age distributions of study populations. When PHN is defined as pain lasting for 3 months, the incidence is 18% in individuals older than 50 years of age and 33% in individuals older than 80 years of age [3].

The development of PHN is associated with increased patient age and severity of acute pain [4]. The diversity of the PHN symptoms (pain, paresthesia, dysesthesia, hyperalgesia, and allodynia) seems to be related to a variety of underlying changes in the nervous system, but this is still unclear. HZ infection is associated with damage to the central and peripheral nervous systems. Although the pathophysiology of PHN is incompletely understood, two possible mechanisms could be responsible: sensitization (peripheral and central neuron generation of spontaneous discharges) and deafferentation (neural damage and inflammation with subsequent edema) [5]. In addition, previous research indicated severe depletion of epidermal free nerve endings in the skin biopsies of patients with PHN, and authors of postmortem studies reported atrophy of the spinal dorsal root ganglia, demyelination with fibrosis, and cell loss [6].

Gabapentin, a structural analogue of γ-aminobutyric acid, has been used for the treatment of PHN for decades, and the results of several randomized controlled trials (RCTs) show that it is a well-tolerated and efficacious treatment in patients with PHN [7]. However, gabapentin used for the prevention of PHN has shown contradictory results. Researchers in an uncontrolled, open-label study reported that administration of gabapentin plus valacyclovir during the acute phase of HZ infection reduced the incidence of PHN [8]. A more recent prospective, controlled, two-armed study using low doses of gabapentin and valacyclovir showed no statistical differences between the two groups regarding PHN prevention [9].

The primary objective of the present study is to evaluate the efficacy of an optimal dose of gabapentin added to the usual treatment—valacyclovir and analgesics as needed—in the prevention of PHN at 12 weeks in patients older than 50 years old who have moderate to severe pain.

## Methods/design

### Design and setting

**Fig. 1:**
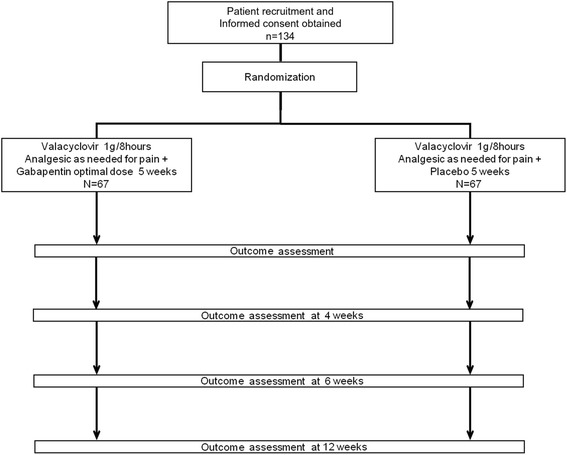
Study time line and flowchart of participants. *IC* Informed consent

**Fig. 2:**
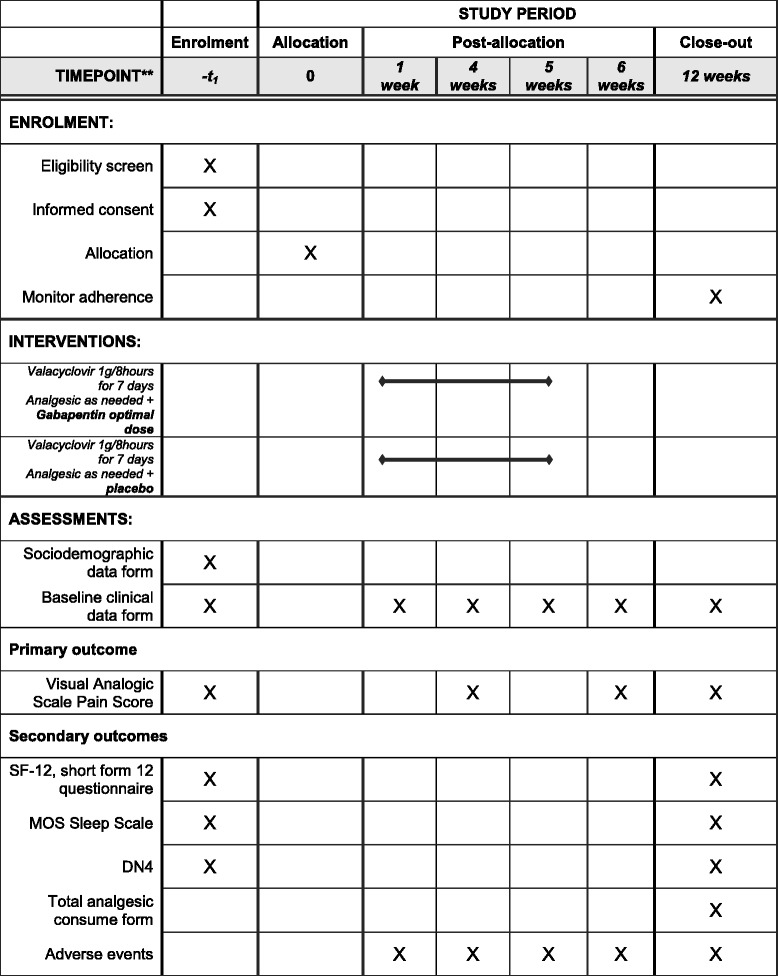
Schedule of enrollment, interventions, and assessments. *MOS* Medical Outcomes Study

We designed a multicenter, parallel, randomized, double-blind, placebo-controlled trial with recruitment of 134 patients from 17 primary care centers in Mallorca, Spain. Participants will be randomly allocated to receive gabapentin or placebo for 8 weeks to evaluate the efficacy of gabapentin in the prevention of PHN. Figure 1 summarizes the study design and time line, and Fig. 2 displays the schedule of enrollment, interventions, and assessments.

### Study population

Male or female patients who are at least 50 years old, have a clinical diagnosis of uncomplicated HZ, present within the first 72 h of vesicle formation, and have an average pain score of at least 4 on a visual analogue scale (VAS) before therapy will be recruited. Table 1 summarizes the eligibility criteria. The study methods are in accordance with the Standard Protocol Items: Recommendations for Interventional Trials (SPIRIT) statement (Additional file 1).

**Table 1:** Patient eligibility criteria for assessment of the efficacy of gabapentin in prevention of postherpetic neuralgia

Inclusion criteria	Exclusion criteria
Male or female, at least 50 years old	Patients taking gabapentin or a tricyclic antidepressant
Patients with diagnoses of uncomplicated herpes zoster presenting within the first 72 h of vesicle formation and an average pain score of at least 4 on a visual analogue scale of pain before therapy	Patients with evidence of cutaneous or visceral dissemination of herpes zoster infection (*cutaneous dissemination* is defined as more than 20 discrete lesions outside adjacent dermatomes) or ocular involvement of herpes zoster
Patients who are willing and able to comply with the requirements of the study	Patients with histories of intolerance or hypersensitivity to any active components of or excipient from the study drugs
Patients who are willing and able to give written informed consent	Patients with severe hepatic impairment or impaired renal function (creatinine clearance <79 ml/minute)
	Patients who have received cytotoxic drugs or immunosuppressive therapy within the previous 3 months (e.g., long-term systemic corticosteroids)
	Patients with any diagnosed immune dysfunction
	Patients who have received immunomodulatory medications (including interferon) within the previous 4 weeks
	HZ vaccine immunization

*HZ* Herpes zoster

**Table 2:** Instruments, assessments, and timing of assessments

Instrument	Assessment	Time of assessment
Sampling form	Inclusion/exclusion criteria	Before randomization
Sociodemographic data form	Sociodemographic data (age, sex, BMI)	Baseline
Baseline clinical data form	Medical history, concomitant medications, and analgesic drugs to control HZ-related pain	Baseline, 1, 4, 6, and 12 weeks
Visual analogue scale pain score	Severity of pain before/after therapy	Baseline, 4, 6, and 12 weeks
SF-12 questionnaire	Health-related quality of life	Baseline and 12 weeks
Monitor adherence	Indirect method: patient questionnaires, patient self-reports, and pill counts	12 weeks
Adverse events	Adverse event evaluations related to study medication	1, 4, 6, and 12 weeks
MOS Sleep Scale	Extent of sleep problems (sleep initiation, maintenance, respiratory problems, quantity, perceived adequacy, and somnolence)	Baseline and 12 weeks
DN4	Neuropathic pain consisting of interview questions and physical tests	Baseline and 12 weeks
Analgesic consumption form	Total consumption of analgesic drugs during the study period	12 weeks
	Number of patients on analgesics at the end of the study	

*Abbreviations: BMI* Body mass index, *DN4* Douleur Neuropathique in 4 Questions, *HZ* Herpes zoster, *MOS* Medical Outcomes Study, *SF-12* 12-item Short Form Health Survey

### Recruitment

Each participating primary care center will have at least one physician from the research team invite potential candidates to participate in the clinical trial and to perform all procedures. All patients diagnosed with acute HZ will be referred to this physician and will be invited to participate in the trial. Willing participants who meet the eligibility criteria will be enrolled after they read and sign an informed consent agreement (Additional file 2).

### Randomization

The central pharmacy of Hospital Son Espases will package the study treatments (placebo or gabapentin) using an unblinded randomization code list. The link between the randomization code and the corresponding treatment will remain blinded for all other study team members. During the process of randomization, each subject will be assigned a randomization code and will be given the treatment package with that code. This sequential randomization will be generated in blocks of six. To assess the effectiveness of masking, patients and investigators will be asked to guess whether they think they received the treatment or placebo at the final visit, and then will be asked to indicate what led them to that belief.

### Intervention

The primary goals in management of HZ are to inhibit ongoing viral replication, alleviate pain, and prevent complications such as local shingles infections or PHN [10]. Local measures to prevent vesicle infection will be recommended to all recruited patients. These measures include frequent hand-washing, drying out of shingles after a shower without scratching the blisters, and covering blisters until they are crusted over.

Antiviral therapy is considered the firstline treatment for HZ and should be initiated within 72 h of onset. Thus, all participants will receive 1000-mg caplets of valacyclovir hydrochloride three times daily for 7 days. The World Health Organization three-step pain relief ladder will be used for pain management. In particular, if pain occurs, there will be prompt oral administration of drugs in the following order, until the patient is free of pain: nonopioids (paracetamol); then, as necessary, mild opioids (codeine); then strong opioids, such as morphine.

### Study medication

Bottles of 300-mg gabapentin capsules and of matching placebos will be dispensed according to the randomization schedule, which is retained by the clinical trial pharmacist. The participants, research staff, and investigators who assess outcomes will be masked to treatment allocations.

Gabapentin will be initiated at 300 mg/day and then increased in a stepwise manner according to the instructions for use. The dose will be increased, regardless of whether efficacy is achieved at a lower dose, to a ceiling daily dose of 1800 mg/day. In patients who develop intolerable adverse effects, the dose will be reduced. The optimal dose established during the titration period will be maintained throughout the remainder of the study and followed by 1 week of dose-tapering. Use of systemic corticosteroids and tricyclic antidepressants will not be allowed.

### Assessing medication adherence

The treatment will be monitored at every visit by the responsible physician at every participating health care center. We will ask the patients to comply with a medical diary that has to be followed until the next appointment or a daily medication box, if wanted. At the last visit, the participants must return their bottles, and a final count will be made by the physician. A biannual study newsletter will be sent to all the researchers, and an annual meeting will take place between the principal investigator and all the study collaborators.

### Data collection methods and record-keeping

Case report forms will be used to record data for all participants. The researchers will receive training for standardized data collection procedure. All data will be stored in a locked cabinet of every researcher until the inclusion period finishes. Patient information will be coded using a unique numerical identification, and the data will be entered into an electronic database and its validity secured. Logical checks will be performed for missing data and to find inconsistencies. The researchers and data analysts will have full access to data.

### Measurement of outcomes

#### ᅟ

Measures and variables are summarized, with a timeline, in Table 2. The main outcome measure will be the incidence of PHN at 12 weeks, defined as an average daily VAS pain score of 0.

#### Secondary outcome variables

##### Response rate

The response rate of the groups at 6 and 12 weeks is a secondary outcome measure. Responders will be defined as those with a 50% reduction in VAS pain compared with baseline. This outcome was chosen following a recommendation from the Committee for Medicinal Products for Human Use in their clinical guidelines on clinical medicinal products intended for the treatment of neuropathic pain [11].

##### Percentage of patients with neuropathic pain (Douleur Neuropathique in 4 Questions)

The change from baseline to the end of the study in the DN4 questionnaire (Douleur Neuropathique in 4 Questions) score will be used to assess changes in neuropathic pain. This scale includes ten items, with each “yes” response scored as 1, and is subdivided into descriptors (seven items) and signs relating to the sensory examination (three items). A score above 4 indicates neuropathic pain. This questionnaire has been validated previously [12, 13], and a validated Spanish version is available [14].

##### Quality of life

The 12-item Short Form Health Survey (SF-12®), a short version of the SF-36®, will be used to assess quality of life. This questionnaire has two questions on physical functioning, two questions on role limitations because of physical health problems, one question on bodily pain, one question on general health perceptions, one question on vitality (energy/fatigue), one question on social functioning, two questions on role limitations because of emotional problems, and two questions on general mental health (psychological distress and psychological well-being). The SF-12 is applicable in different cultures, and a validated Spanish version is available [15, 16].

##### Sleep interference

The Medical Outcomes Study Sleep Scale will be used to assess the quality and quantity of sleep. This questionnaire has 12 items that assess the key constructs of sleep. It is self-administered, and patients are asked to recall sleep-related activities over the past 4 weeks. It comprises scoring in six domains: sleep disturbance (four items), snoring (one item), awakening with shortness of breath or a headache (one item), quantity of sleep (one item), optimal sleep (one item), sleep adequacy (two items), and daytime somnolence (three items). This scale has been validated in Spanish for patients with neuropathic pain [17].

##### Patient Global Impression of Change Scale

The Patient Global Impression of Change Scale comprises a single question in which the patient is asked to rate his/her present condition relative to how it was prior to treatment on a scale from 1 (very much better) to 7 (very much worse). This scale has been used in previous studies in which researchers assessed patients’ impressions of improvement following PHN treatment [18].

##### Analgesic consumption

Medications taken for control of pain will be recorded at baseline and at every follow-up visit during the study period.

### Safety

Patients will be interviewed at each study visit regarding the occurrence of any adverse events (AEs), including type of event, time of onset, duration, and severity. Safety analyses will be performed on the safety population, which will consist of all patients who received at least one dose of the study drug. Safety data will include the incidence of treatment-emergent AEs, serious adverse events (SAEs), and the number and percentage of patients reporting one or more AEs in each group.

### Withdrawals

Participants will be free to withdraw from participation at their own request at any time without giving reasons for their decision. Withdrawals will be documented in the case report forms and in patients’ medical records, with active follow-up for ongoing SAEs.

### Adverse effects

All information regarding AEs will be presented in the case report form. The study investigators will investigate the causal relationship of the study drug and the intensity of AEs. Any SAE (e.g., death, a life-threatening event, inpatient hospitalization or prolongation of existing hospitalization, persistent or significant disability/incapacity) in any patient during the course of the study will be reported to the ethics committee [19].

### Sample size

The sample size calculation is based on the primary outcome measure and the primary analysis for the intention-to-treat population. Researchers in a previous nonrandomized, noncontrolled experimental study reported the incidence of PHN was 20% [8]. Thus, we estimated a 45% incidence of PHN in the placebo group, based on its incidence in patients older than 50 years old with an average VAS pain score of 4 or more reported in a longitudinal study [20]. We adjusted the sample size for an estimated follow-up loss rate of 20% and a 0.05 two-sided level of significance (α = 5%). Thus, we will need 67 patients in each group to detect a difference of at least 25% in the incidence of PHN in the treatment and placebo arms.

### Statistical analysis

We will test for significant baseline differences in the placebo and gabapentin arms by use of descriptive analysis, with continuous variables summarized by means and SDs for normal distributions and by medians and 25th and 75^th^ percentiles for nonnormal distributions. All analyses of the effectiveness and cost-effectiveness will involve intention-to-treat populations (i.e., all randomized patients, regardless of participation in any treatment session). This approach reduces the bias that may occur when participants not receiving assigned treatments are excluded from analysis. All tests will be two-sided, and an α value of 0.05 will be considered statistically significant.

We will compare the proportions of patients in each arm with PHN at 12 weeks against the null hypothesis of no difference between the groups. We will use the chi-square test in multivariate analysis and will adjust for potential confounders, if any, using a logistic regression model. We will estimate relative and absolute risk reduction and the number needed to treat, defined as the estimated number of patients who need to be treated with gabapentin for prevention of PHN in one patient.

The health economic analysis will be performed by calculating the incremental cost-effectiveness ratio (ICER) at 12 weeks. We will systematically collect data on use of all resources, including inpatient care, consultations with health care providers, use of drugs, and laboratory tests. To measure effects, the SF-12 scores will be transformed into EQ-5D utility scores and quality-adjusted life-years (QALYs) will be determined. The ICER will be calculated as the difference in the mean costs of the two groups divided by difference in the mean effects of the two groups:

$$ ICER=\frac{{\overline{C}}_I-{\overline{C}}_T}{{\overline{E}}_I-{\overline{E}}_T} $$

A nonparametric bootstrap procedure will be used to perform the uncertainty analysis for the ICER. This procedure considers the skewness of cost data and the covariance of costs and QALYs. To control for possible confounding variables and to account for clustering, an alternative procedure (net-benefit regression) will also be used. Cost-effectiveness acceptability curves will be created to illustrate statistical uncertainty. We will determine the safety of interventions in the safety population and use per-protocol analysis with the chi-square test by comparing the AEs among patients.

All estimates will include 95% confidence intervals. The number needed to treat will be calculated as the reciprocal of the difference between the proportion of patients with PHN in the placebo and gabapentin arms.

### Approval

This study will follow the principles outlined in the Declaration of Helsinki. All patients will be asked to provide written informed consent and will be told that participation is voluntary and can be withdrawn at any time without any negative consequences concerning their current or future medical treatments. Our study protocol has been approved by the Primary Care Research Committee, the Mallorca Ethical Committee of Clinical Research (IB 1857/12), and the Spanish Agency on Drugs and Medical devices (for Agencia Española de Medicamentos y Productos Sanitarios). Any protocol modification will be approved by the executive committee, submitted to the ethics committee for approval, and noted to the ISRCTN registry. Trial participants will be notified if relevant protocol changes will be made.

## Discussion

PHN is a persistent nerve pain that has an adverse effect on quality of life in patients with HZ. It has a high prevalence among patients with HZ who are older than 50 years old and in those with moderate to severe pain, although previous studies have used different definitions of PHN. The definition of PHN in the proposed study is persistent pain for more than 90 days since the onset of shingles, considered the most accepted definition for PHN [21, 22].

Treatments for PHN attempt to alleviate the pain, and several pharmacological strategies are available. This includes tricyclic antidepressants, anticonvulsants, analgesics, and topical agents. However, no disease-modifying therapy is available [23], and preventive strategies are urgently needed. Preventive strategies such as varicella zoster virus live-attenuated vaccine (approved by the U.S. Food and Drug and Administration for adults older than 50 years old) showed reductions of the incidence of HZ infection and PHN [22], but RCTs are needed to confirm these results.

Gabapentin acts on supraspinal region to stimulate noradrenaline-mediated descending inhibition [24–26]. We hypothesize that preventing central sensitization in patients with HZ will reduce the incidence of PHN.

Thus, gabapentin may provide pain relief, but whether pain relief could also prevent the onset of PHN is not yet clear. In our study, participants in both arms will receive analgesic treatments as needed to provide pain relief. There is a significant increase in the risk of PHN following acute zoster infection, including prodromal pain and severe rush. Thus, participants in both of our study arms will also receive valacyclovir within 72 h after rash appearance. Valacyclovir is preferred over other antivirals because it more easily produces consistently high levels in the blood, patient compliance is better, and less frequent dosing is required.

The effectiveness of gabapentin for the prevention of NPH was previously evaluated in two studies. Researchers in an uncontrolled open-label study concluded that the combination of gabapentin and valacyclovir reduces the incidence of PHN [8]; however, although the gabapentin dose could be titrated up to 3600 mg/day, only a few patients reached that dose, and the median dose was 1085 mg/day for 34 days. This study was criticized because there was no control group; instead, the authors compared the incidence of PHN with historic control subjects described in a meta-analysis of six RCTs of antiviral agents used to treat acute zoster infection [27]. These authors also concluded that larger-scale blinded studies are necessary to confirm their results.

A prospective controlled study showed that low doses of gabapentin were not effective in the prevention of PHN [9]. However, the study was nonrandomized, and patients were not blinded to treatment.

The two main strengths of our study are that, as far as we know, it is the first RCT to examine the effect of gabapentin on prevention of PHN, and it is an independent clinical trial funded by a public research agency. The ceiling dose of gabapentin is 1800 mg/day because it has been established that a dose greater than 1800 mg/day does not generally provide greater benefit; the bioavailability of gabapentin varies inversely with dose, and high-dose regimens are associated with lower patient compliance [28]. Gabapentin is compared with placebo treatment because there is no convincing evidence that other treatments can reduce PHN after HZ has been established. Currently, the management of HZ and PHN is based largely on general practice. This RCT is developed entirely in a primary care setting and will have the participation of 17 primary care teams. The external validity will be assured because the effectiveness of gabapentin treatment in the prevention of PHN will be assessed in the same settings in which most HZ cases are treated.

Limitations of our study include the possibility of a high discontinuation rate due to gabapentin’s potential side effects, possible interactions of gabapentin with other drugs, and the complex dosage regimen. The most recognizable side effects of gabapentin are dizziness, somnolence, and drowsiness, so their presence might compromise the blinding of patients and investigators. Although we considered the use of an active placebo (a placebo that mimics the side effects of the drug under evaluation), we finally decided to use a “pure” placebo because of ethical considerations for the patients included in the study.

HZ and PHN have major impacts on patients’ lives [29] and constitute a significant economic burden for health care systems and societies at large [30]. A treatment that effectively prevents PHN in patients at high risk could improve the quality of life of patients with HZ and also reduce health care costs.

### Trial status

At the time of this writing, 70% of the target population of 134 has been enrolled. The anticipated study completion date is January 2017.

## Additional files

Additional file 1: SPIRIT checklist. (PDF 41 kb)
Additional file 2: Model consent form. (DOC 38 kb)
