# Supplementary material for: Efficacy of gabapentin for prevention of postherpetic neuralgia: study protocol for a randomized controlled clinical trial
Source: Trials. 2017 Jan 14;18:24. doi: 10.1186/s13063-016-1729-y (PMC5237496; doi:10.1186/s13063-016-1729-y)
Supplement: Additional file 2: — Model consent form. (DOC 38 kb) [file 13063_2016_1729_MOESM2_ESM.doc]

**HOJA DE INFORMACIÓN AL PACIENTE**

**TÍTULO DEL ESTUDIO:** Ensayo clínico, aleatorizado, doble-ciego para evaluar la eficacia y seguridad de la gabapentina frente a placebo en la prevención de la neuralgia postherpética

**CÓDIGO DEL PROMOTOR**: PI12_01813

**PROMOTOR:** Gerencia de Atención Primaria Mallorca

**INVESTIGADOR PRINCIPAL**: Manuel Rullán García

**CENTRO:** CS Pollença

**INTRODUCCION**

Nos dirigimos a usted para informarle sobre un estudio de investigación en el que se le invita a participar. El estudio ha sido aprobado por el Comité de Ética de la Investigación de las Illes Balears y la Agencia Española del Medicamento y Productos Sanitarios, de acuerdo a la legislación vigente, y se lleva a cabo con respeto a los principios enunciados en la declaración del Helsinki y a las normas de buena práctica clínica.

Nuestra intención es tan solo que usted reciba la información correcta y suficiente para que pueda evaluar y juzgar si quiere o no participar en este estudio. Para ello lea esta hoja informativa con atención y nosotros le aclararemos las dudas que le puedan surgir después de la explicación. Además, puede consultar con las personas que considere oportuno.

Debe saber que su participación en este estudio es voluntaria y que puede decidir no participar o cambiar su decisión y retirar el consentimiento en cualquier momento, sin que por ello se altere la relación con su médico ni se produzca perjuicio alguno en su tratamiento.

En algunos casos y dependiendo de la edad, las personas que han sufrido un herpes zoster desarrollan dolor en la zona afectada con diferente intensidad, se piensa que entre un 15 y un 50% dependiendo de la edad.

El ensayo clínico al que se le invita a participar pretende determinar si la administración de gabapentina en las primeras 72h del inicio del herpes zoster y durante 5 semanas reduce el número de personas que sufren dolor después del episodio de herpes zoster, ni el médico ni usted sabrán cuál es el tratamiento que va a recibir, para ello la mitad de los pacientes recibirán el tratamiento con gabapentina y la otra mitad recibirán una capsula con las mismas características que la de gabapentina pero que no contiene sustancia farmacológicamente activa, igual que si usted se tomara un caramelo.

El estudio tiene una duración de 12 semanas, usted tendrá que acudir a 4 visitas con su médico y una visita realizada con una persona del estudio. En este estudio participarán 134 pacientes.

Si usted recibe el tratamiento no activo, como si fuera un caramelo, no recibirá ni beneficio ni perjuicio por participar en el estudio, si usted recibe el tratamiento activo (gabapentina) y los resultados son positivos, usted y otras personas con herpes zoster pueden que no desarrollen dolor en la zona afectada después de haber sido tratada, aunque también es posible que no obtenga ningún beneficio para su salud, también queremos informarle que aproximadamente la mitad de los pacientes que tomen el tratamiento activo serán tratados aunque su dolor hubiera desaparecido espontáneamente antes de las 12 semanas en que finalizará el estudio*.*

La gabapentina es un fármaco comercializado y se utiliza para el tratamiento de la epilepsia y del dolor neuropatico, en los estudios de este fármaco han demostrado que es un fármaco que puede ser utilizado con seguridad en personas, sin embargo en estos estudios algunas personas desarrollaron efectos no deseados como: somnolencia, mareos vértigo e hinchazón en las extremidades.

La gabapentina como todos los agentes anticonvulsivantes actúa sobre el sistema nervioso central y puede producir somnolencia, vértigo, u otros síntomas relacionados. Aunque sólo sean de intensidad leve o moderada, estos efectos adversos pueden ser potencialmente peligrosos para los pacientes que conducen o manejan maquinaria, particularmente hasta el momento en que la experiencia individual del paciente esté consolidada.

Debe aconsejarse a los pacientes que se abstengan de conducir o manejar maquinaria hasta que se conozcan los efectos de gabapentina, así mismo la ingesta de alcohol o fármacos puede agravar algunos de los efectos secundarios de gabapentina relacionados con el SNC p. Ej. somnolencia, ataxia.

Usted no tendrá que pagar por los medicamentos del estudio.

Su médico recibirá una compensación económica por su participación en este estudio y ha declarado si existe o no conflicto de intereses.

El tratamiento, la comunicación y la cesión de los datos de carácter personal de todos los sujetos participantes se ajustará a lo dispuesto en la Ley Orgánica 15/1999, de 13 de diciembre, de protección de datos de carácter personal, y en su reglamento de desarrollo. De acuerdo a lo que establece la legislación mencionada, usted puede ejercer los derechos de acceso, modificación, oposición y cancelación de datos, para lo cual deberá dirigirse a su médico del estudio.

Sus datos serán tratados informáticamente y se incorporarán a un fichero automatizado de datos de carácter personal cuyo responsable es la Gerencia de Atención Primaria, que ha sido registrada en la Agencia Española de Protección de Datos.

Sus datos recogidos para el estudio estarán identificados mediante un código y solo su médico del estudio y colaboradores podrán relacionar dichos datos con usted y con su historia clínica. Por lo tanto, su identidad no será revelada a persona alguna salvo en caso de urgencia médica o requerimiento legal.

Sólo se transmitirán a terceros y a otros países, previa notificación a la Agencia Española de Protección de Datos, los datos recogidos para el estudio que en ningún caso contendrán información que le pueda identificar directamente, como nombre y apellidos, iniciales, dirección, nº de la seguridad social, etc. En el caso de que se produzca esta cesión, será para los mismos fines del estudio descrito y garantizando la confidencialidad como mínimo con el nivel de protección de la legislación vigente en nuestro país.

El acceso a su información personal quedará restringido al médico del estudio/colaboradores, autoridades sanitarias, al Comité de Ética de la Investigación de las Illes Balears y personal autorizado, cuando lo precisen para comprobar los datos y procedimientos del estudio, pero siempre manteniendo la confidencialidad de los mismos de acuerdo a la legislación vigente.

También debe saber que usted puede ser retirado del estudio en caso de que los responsables del estudio lo consideren oportuno, ya sea por motivos de seguridad, por cualquier acontecimiento adverso que se produzca por la medicación en estudio o porque consideren que no está cumpliendo con los procedimientos establecidos. En cualquiera de los casos, usted recibirá una explicación adecuada del motivo que ha ocasionado su retirada del estudio.

Si usted es retirado del estudio, por alguno de los motivos expresados, su médico le prescribirá un tratamiento adecuado a su enfermedad.

Al firmar la hoja de consentimiento adjunta, se compromete a cumplir con los procedimientos del estudio que se le han expuesto.

Para cualquier duda se puede poner en contacto con:

Unidad de Investigación Gerencia de atención Primaria de Mallorca:

Telf 971 175884

**CONSENTIMIENTO INFORMADO**

Caso nº: ____________

Título del ensayo:

***Eficacia comparativa de dos intervenciones del médico de familia para el abandono del consumo crónico de benzodiacepinas.***

Yo, _________________________________________________________

(Nombre y apellidos)

1. He leído la hoja de información del estudio.
2. He podido hacer preguntas sobre el estudio.
3. He recibido suficiente información sobre el estudio.
4. He hablado con ___________________________________

(Nombre médico investigador)

1. Comprendo que mi participación es voluntaria.
2. Se que puedo retirarme del estudio cuando quiera, sin tener que dar explicaciones y sin que esto repercuta en mis atenciones médicas.

Doy libremente la conformidad para participar en el estudio.

Firma del paciente Firma del médico responsable

Nombre del Paciente______________ Nombre del médico_________

Fecha: Fecha:

Investigador prinicipal del estudio:

Manual Rullán García

CS Pollença.

Tlf: 971533041

[mrullan@ibsalut.caib.es](mailto:mrullan@ibsalut.caib.es)
